# Nutritional Strategies to Mitigate Post-Weaning Challenges in Pigs: A Focus on Glucans, Vitamin D, and Selenium

**DOI:** 10.3390/ani14010013

**Published:** 2023-12-19

**Authors:** John O’Doherty, Alison Dowley, Eadaoin Conway, Torres Sweeney

**Affiliations:** 1 School of Agriculture and Food Science, University College Dublin, Belfield, D04 W6F6 Dublin, Ireland; alison.dowley@ucdconnect.ie (A.D.); eadaoin.conway@ucdconnect.ie (E.C.); 2 School of Veterinary Medicine, University College Dublin, Belfield, D04 W6F6 Dublin, Ireland; torres.sweeney@ucd.ie

**Keywords:** pig, weaning, β-glucans, mushrooms, Vitamin D, selenium, dam

## Abstract

**Simple Summary:**

The pig-farming industry faces significant challenges in ensuring the health and growth of piglets, particularly during the weaning phase. This critical period involves multiple stressors, such as environmental changes, dietary shifts, and social separation, which can adversely affect the piglet’s digestive health, immune system, and overall well-being. One of the primary hurdles during weaning is the transition from a milk-based diet to a more complex cereal-based diet. This abrupt dietary change can lead to reduced food intake, digestive issues, gut inflammation, and nutrient absorption difficulties, resulting in diarrhea and poor growth. To tackle these issues, researchers are exploring innovative nutritional strategies. One promising area is the utilization of specific types of fiber, known as glucans, derived from sources like cereals, mushrooms, seaweed, and yeast. Additionally, there is a growing focus on the roles of Vitamin D and selenium, with Vitamin D and selenium-enriched mushrooms serving as natural sources of these vital nutrients. In conclusion, addressing the challenges faced by piglets during weaning necessitates the development of effective nutritional strategies, including the incorporation of glucans, Vitamin D, selenium, and enriched mushrooms. These approaches align with sustainable and responsible pig-farming practices, prioritizing the welfare of the animals and reducing the need for additives and antibiotics.

**Abstract:**

This review examines the challenges faced by the pig industry, with a specific focus on improving the health and growth of weaned pigs. It emphasizes the immediate necessity of investigating alternative approaches to managing pig nutrition and health due to restrictions on the use of antibiotics and the prohibition of zinc oxide in weaned pig diets. The weaning phase is identified as a critical stage in piglet development, characterized by stressors that affect their gastrointestinal health, immune responses, and overall physiology. The primary challenge during weaning arises from transitioning piglets from a digestible milk-based diet to a less digestible cereal-based feed, causing nutritional stress. This manifests as reduced feed intake, leading to gastrointestinal disturbances, intestinal inflammation, and adverse effects on intestinal structure and microbiota. To address these challenges and optimize piglet development, various nutritional strategies have been explored. Notably, glucans, particularly β-glucans from fungi, cereals, algae, and yeast, show promise in alleviating weaning-related issues. Furthermore, it is important to highlight the critical roles played by Vitamin D and selenium in piglet nutrition. These essential nutrients can be sourced naturally from enriched mushrooms that are specifically enriched with Vitamin D and selenium, providing a sustainable dietary option. In conclusion, effective nutritional strategies, including glucans, Vitamin D, selenium, and enriched mushrooms, are beneficial for addressing weaning-related challenges.

## 1. Introduction

Weaning is a critical phase in the development of piglets, with profound effects on their gastrointestinal health, immune responses, and overall physiology [1]. This stage introduces a complex interplay of stressors, including environmental shifts, dietary changes, and social separations, all of which collectively impact production efficiency [2,3]. The nutritional transition during weaning, which involves a shift from a milk-based to a cereal-based diet, poses a significant challenge to the piglet’s digestive capacities [4]. Additionally, the move from farrowing rooms to weaner houses exposes them to novel environments and increased pathogen exposure. Social stress is further intensified as piglets are abruptly separated from their mothers and integrated with new piglets. These multifaceted stressors result in decreased feed intake and a range of gastrointestinal issues [5]. In contrast, wild piglets undergo a gradual weaning process over 10–14 weeks, allowing for more robust gastrointestinal development [6]. Commercial weaning, typically occurring between 3 and 4 weeks, coincides with the peak development of the gastrointestinal barrier but leaves the piglet’s immune systems underdeveloped. Delaying weaning can enhance disease resistance but comes at the cost of increased production costs.

Following weaning, piglets undergo significant alterations in intestinal morphology, which affect nutrient absorption [7,8]. These changes are linked to an increased incidence of post-weaning diarrhea (PWD) and reduced enzymatic activity, which is essential for nutrient digestion [9]. Consequently, disruptions in the intestinal barrier lead to heightened immune system responses [10]. The weaning process triggers inflammation in piglets due to their immature immune systems and exposure to various antigens [11].

To address these intricate issues, various nutritional strategies have been explored in piglet rearing to enhance health and growth. Glucans, specifically β-glucans derived from sources like seaweed, mushrooms, cereals, and yeast, have gained attention for their potential to alleviate some of the weaning-related challenges. Additionally, the roles of Vitamin D and selenium, two essential nutrients, are emerging as important factors in piglet nutrition. Casein hydrolysates, which are derived from milk protein casein, are also gaining attention as potential alternatives to antibiotic growth promoters in pig nutrition. Understanding the underlying physiological changes during this critical phase is vital for developing effective nutritional strategies. Until recently, the main focus on finding alternatives to in-feed antibiotic growth promoters and zinc oxide has been on dietary manipulations in pigs post-weaning using feed additives in the post-weaning diet. However, other strategies, such as maternal nutrition to improve growth and health in her offspring, also hold promise. This review aims to delve into these strategies, with a focus on glucans, casein hydrolysates, Vitamin D, and selenium, and assess their potential to mitigate the complexities of weaning-related challenges.

## 2. The Physiological and Immunological Implications of Weaning on Piglets

Weaning is a critical period characterized by considerable stress, influencing the piglet’s gut microbiology, immunology, and physiology, which in turn impacts growth, intake, and health [3]. Environmental changes, dietary shifts, and social reorganization contribute to this stress. Unlike the gradual weaning process observed in the wild, commercial weaning procedures are abrupt, potentially increasing health risks due to the piglet’s reliance on maternal antibodies [12]. During the suckling period, the piglet’s gut microbiota is significantly influenced by the constituents of milk, which promotes the growth of bacterial families such as *Bacteroidaceae*, *Clostridicaceae*, *Lachnospiraceae*, and *Lactobacillicaeae* [13]. However, with the introduction of solid feed and cessation of milk at weaning, substantial changes occur in the intestinal microbiota composition, demonstrated by the emergence of substantial shifts in the population dynamics of gut bacteria [14]. The weaning transition, coupled with a decline in lactose availability, results in higher gastric pH levels, reducing the natural barrier to enteric infections [1,15]. Concomitantly, there is a decrease in beneficial microbes such as *Lactobacillus*, alongside an increase in opportunistic pathogens, including *Clostridium* and *E. coli*, the latter of which plays a pivotal role in the etiology of PWD [16,17]. As the piglet matures, the GIT microbiota diversifies and stabilizes, approximating an adult-like composition by three weeks post-weaning, characterized by a higher abundance of *Prevotellaceae*, *Ruminococcaceae*, *Lactobacillaceae* and *Veillonellaceae*, with a concurrent reduction in *Enterobacteriaceae* and *Bacteroidaceae* [18].

Following weaning, intestinal morphological changes, such as fluctuations in villous height and crypt depth, affect the organ’s absorptive function and overall health [19]. Optimal nutrient uptake is compromised by the reduced activity of digestive enzymes such as lactase, sucrase, and maltase, which are critical for carbohydrate digestion [7,20]. These physiological alterations are further aggravated by disruptions to the intestinal barrier, mediated by the inflammatory response, and characterized by weakened tight junctions between epithelial cells [10,21]. Additionally, stress mediators, including cortisol and corticotropin-releasing factors, have been implicated in neuro–immune interactions that adversely affect gastrointestinal functionality [6]. Post-weaning also sees a marked reduction in feed intake, with subsequent negative repercussions for gastrointestinal health and growth [7]. Furthermore, PWD, primarily caused by *E. coli*, poses a significant challenge in pig production. The enterotoxigenic strains of this bacterium bind to the intestinal epithelium, facilitated by fimbriae such as F4 and F18, leading to infection and diarrheal outbreaks [22,23]. Various management strategies that focus on nutrition and the weaning process itself are critical for minimizing these episodes [17]. In summary, weaning represents a developmental stage with profound implications for the gastrointestinal health of piglets. Effective management of this phase, including the understanding of associated stressors and implementation of nutritional interventions, is essential to promote health and growth in piglets, with the added benefit of reducing the need for medicinal supplements such as zinc oxide and in-feed medication in pig production.

## 3. Exploring Natural Dietary Interventions to Address Dysbiosis in Pig Nutrition

This review delves into the evolving dynamics and challenges within the pig industry, placing a strong emphasis on advancing the health and growth of weaned piglets. A central theme of the review revolves around exploring natural compounds and nutrients as potential dietary supplements for pigs, with a particular focus on the advantages associated with incorporating β-glucans, casein hydrolysates, mushrooms, Vitamin D, and selenium and their synergisms into piglet and sow diets. These natural compounds are acknowledged for their multifaceted properties, encompassing anti-inflammatory, antimicrobial, antioxidant, immunomodulatory, and prebiotic effects. The review emphasizes the major role of maternal nutrition in shaping the health and well-being of piglets, examining how maternal dietary interventions significantly impact piglet development and resilience, both before and after weaning. Another aspect of the review lies in its examination of the unique capabilities of mushrooms, specifically their ability to synthesize Vitamin D and convert inorganic selenium into highly bioavailable organic forms. This positions mushrooms as invaluable additions to pig diets, presenting opportunities to enhance piglet health and performance post-weaning. The review stresses the importance of understanding the underlying mechanisms governing the functional properties of these feed ingredients. Key aspects of gut functionality, including digestive and absorptive capacity, villi architecture, nutrient transporter expression, chemical and physical barriers, microbiota diversity, and immune function, are considered to achieve an optimal response to these dietary interventions [11,24].

## 4. Structural Specificity and Health Impact of Beta-Glucans in Pig Nutrition

The role of β-glucans in pig nutrition is increasingly recognized for its profound impact on enhancing the immune response and overall health of pigs. These naturally occurring polysaccharides, comprised of glucose molecules linked by β-glycosidic bonds, display a structural diversity that is crucial to their functionality. Depending on their botanical or microbial origin, ranging from cereals like barley and oats to yeasts and mushrooms—the arrangement of these linkages can be primarily 1,3, 1,4, or 1,6, each conferring different physical properties and biological activities. For example, the β-glucans from mushrooms have a 1,3 backbone with 1,6-linked side chains, a structure that has been shown to potentiate immune response through various mechanisms, including the activation of macrophages and other immune cells [25]. Yeast-derived β-glucans, with a higher proportion of 1,3 linkages, have been found to enhance pathogen recognition by the immune system [26]. Conversely, the β-glucans found in cereals, primarily with 1,3 and 1,4 linkages, exhibit a solubility that can influence gut health, which in turn can have a systemic effect on the immune status of pigs [27]. This specificity necessitates a nuanced approach to dietary inclusion, where factors like source, purity, solubility, synergisms, and molecular mass must be carefully considered to ensure the β-glucans incorporated into pig feeds are optimized for the best possible health outcomes [28]. In essence, the structural complexity of β-glucans dictates their potential as a functional feed additive in pig nutrition.

Importantly, all three types of β-glucans have been linked to improved growth performance and feed efficiency in pigs, operating through distinct mechanisms. Yeast and seaweed-derived β-glucans likely enhance growth by boosting health and disease resistance, while cereal-derived β-glucans improve growth through better gut health and nutrient absorption. The selection of β-glucan source should be guided by specific production goals, like enhancing immune function, improving gut health, or optimizing growth performance in pigs.

## 5. Yeast-Derived β-Glucans: Enhancing Swine Nutrition and Health during Weaning

### 5.1. Immunomodulatory Potential of Yeast-Derived β-Glucans

β-Glucans, a class of vital polysaccharides, have gained increasing recognition for their health-promoting roles in functional foods and animal nutrition. These molecules are diverse and can be found in various sources, including bacteria, fungi, algae, and cereals, each displaying unique structural attributes. Yeast-derived β-glucans are characterized by having β (1,6)-linked branches on a β (1,3) backbone, in contrast to the primarily linear β (1,4) linkages interspersed with β (1,3) chains typically found in cereal β-glucans [25]. One of the prominent features of yeast-derived β-glucans is their capacity to stimulate the immune system, as they activate a wide range of immune cells, including macrophages, T helper cells, and natural killer cells [29]. This immune activation occurs through their interaction with pattern recognition receptors, which identify these polysaccharides as pathogen-associated molecular patterns, therefore triggering innate immune responses [30,31]. Among these receptors, Dectin-1 plays a crucial role in recognizing β-glucans and initiating immune responses, encompassing processes such as phagocytosis, oxidative burst, and cytokine production [32,33,34].

The immunomodulatory activity of β-glucans is significantly influenced by their structure and solubility. Insoluble β-glucans activate the dectin-1 pathway, while soluble forms often interact with the complement system, with their action being dependent on specific antibodies [33]. The effectiveness of dectin-1 receptor activation by β-glucans requires a β (1,3) backbone of sufficient length, typically consisting of at least seven glucose units, and often necessitates at least one β (1,6)-side-chain branch [33].

### 5.2. Enhancing Growth Performance with Yeast-Derived β-Glucans

In the field of pig nutrition, β-glucans, particularly those derived from yeast, have been associated with improved growth performance in weaned pigs. Multiple studies have demonstrated that β-glucans sourced from organisms like *Agrobacterium* spp. and yeast can positively influence the gut microbiota, increasing the abundance of beneficial bacterial taxa such as *Lactobacillus* while reducing harmful species like *E. coli* [28,35,36,37,38,39]. Moreover, yeast β-glucans are known for their anti-inflammatory and antioxidant properties, which may provide added resilience against chronic inflammation and oxidative stress. A recent meta-analysis [40] quantified these benefits, revealing a 7.6% increase in weight gain and a 5.3% increase in feed intake among nursery pigs fed with yeast β-glucans sourced from Saccharomyces cerevisiae. Even more substantial growth improvements were observed with Agrobacterium-derived β-glucans. The meta-analysis recommended an optimal use of 50 mg/kg of Saccharomyces cerevisiae-derived β-glucans in nursery pig diets based on these findings [40].

Kim et al. [39] observed that dietary β-glucans led to a substantial improvement in the daily gain of weaned pigs, with this improvement attributed to enhanced nutrient absorption and improved intestinal health. These findings align with previous research [37], which noted an enhancement in feed conversion ratios with β-glucan supplementation. The pivotal role of β-glucans in promoting gut health has been well emphasized [41]. β-glucans contribute to maintaining the integrity of the gut barrier and support the establishment of a healthy gut microbiome [41], which is critical for preventing post-weaning diarrhea. Additionally, β-glucans also have the potential to alleviate weaning stress in pigs [40]. This study revealed that β-glucans reduced stress-related behaviors and improved feed intake in post-weaned pigs, a factor crucial for ensuring steady growth during the post-weaning period. Research on β-glucans from Agrobacterium sp. ZX09, known for its high purity compared to yeast, has shown positive effects on weaned piglet growth and intestinal health [42]. Both low and high molecular weight β-glucans from *Agrobacterium* sp. ZX09 enhanced piglet growth, emphasizing the importance of source and purity. Low molecular weight β-glucans had strong antioxidant effects, improved mucosal barrier function, and positively affected gut microbiota. High molecular weight β-glucans specifically benefited hindgut bacteria [42].

In summary, current research provides compelling evidence for the significant advantages of incorporating microbial β-glucans, particularly those derived from yeast and bacteria, into the diets of nursery pigs. These β-glucans contribute to enhanced growth, intestinal health, and immune function, ultimately resulting in improved growth performance and overall well-being.

## 6. The Role of Cereal-Derived β-Glucans in Piglet Diets

### 6.1. Impact on Gastrointestinal Microbiota

Cereal β-glucans have a significant impact on the gastrointestinal microbiota of pigs, enhancing beneficial bacteria populations such as *Lactobacillus* and *Bifidobacteria*, which are vital to pig health [43]. These polysaccharides also modulate the cycling of nutrients within the pig’s system, as indicated by their effect on the excretion patterns of urinary and fecal nitrogen, suggesting improved nutrient utilization [44]. Moreover, β-glucans have been shown to contribute to the reduction of emissions from pig manure, aligning pig-farming practices with environmental sustainability goals [45,46]. However, the inclusion of high levels of intact β-glucans may impede nutrient digestibility, potentially affecting the economic efficiency of pig nutrition [44]. Studies by Metzler and Zebeli [46] have correlated high β-glucan content with a decrease in the apparent ileal digestibility and the total tract digestibility of crude protein and energy, highlighting the necessity for careful dietary inclusion to prevent negative effects on nutrient utilization. At optimal concentrations, cereal β-glucans can increase caecal volatile fatty acids and butyrate levels, conferring a gut health advantage [46]. However, their water-soluble nature can also lead to increased digesta viscosity, which may interfere with feed digestion and nutrient absorption, a concern particularly relevant to the growth and health of nursery pigs [46].

### 6.2. Structural Configurations and Physiological Impact

The structural configurations of β-glucans, especially the ratios of β (1,3) to β (1,4) linkages, are key determinants in their physicochemical properties, influencing their solubility and the extent of microbial degradation in the pig’s gastrointestinal tract [47]. A higher ratio of β (1,3) linkages is known to increase solubility and viscosity, impacting the digestive process. The concentration and solubility of β-glucans differ among cereal grains; for instance, barley has a higher β-glucan content in its endosperm and is characterized by a greater proportion of water-insoluble β-glucans compared to oats [48].

Meta-analytic insights indicate inconsistent growth and feed intake responses to dietary cereal β-glucans [40], suggesting that while certain levels can promote intestinal health, demonstrated by prebiotic effects that bolster beneficial gut microbiota and reduce pathogenic bacteria adhesion, the responses are not uniform. The source of β-glucans plays a significant role; oat-derived β-glucans affect gut microbiota differently from barley-derived β-glucans [48,49]. Therefore, formulating pig diets with β-glucans requires a strategic approach that considers both the type and amount, particularly in cases of soluble β-glucans from barley that could heighten digesta viscosity and associate with digestive issues such as PWD [40]. By contrast, the research of Bach Knudsen and Jørgensen [50] underlines the importance of soluble fiber β-glucans in improving the gut environment of weaned pigs. These fibers increase the viscosity of gut contents, which slows the passage rate and facilitates better nutrient absorption, which is beneficial during the weaning phase when pigs are prone to nutritional upsets and often exhibit inefficient digestion. Therefore, the formulation of pig diets with cereal β-glucans calls for a strategic approach that weighs the type and amount, particularly in the case of soluble β-glucans from barley that could increase digesta viscosity and be associated with digestive issues such as PWD [40].

## 7. Seaweed: A Sustainable β-Glucan Source for Swine Nutrition

### 7.1. Immunomodulatory Benefits of Algae β-Glucan

Algae-derived β-glucans are attracting increasing attention as a promising dietary supplement for weaned pigs, offering unique advantages for both growth and health during this crucial developmental stage. One of the primary advantages of incorporating algae β-glucans into the diets of piglets lies in their substantial immunomodulatory capabilities. Algae-derived β-glucans play a vital role in boosting the immune response of weaned pigs [24,51]. This enhancement of the immune system is of paramount importance as it contributes to reducing the occurrence of common post-weaning issues such as diarrhea and respiratory infections. By fortifying the immune defenses, algae β-glucans provide piglets with increased resilience against the microbial threats they encounter in the post-weaning phase [24].

### 7.2. Laminarin: Antibacterial and Prebiotic Effects

Laminarin, a low molecular weight β-glucan found in various seaweeds, is particularly noteworthy for its antibacterial activity. It is characterized by a linear backbone of (1,3)-β-linked glucopyranose residues with varying β-(1,6)-branching [52,53]. The water solubility of laminarin is influenced by its branching levels, and it accumulates in algal cells during specific seasons to support survival and growth during adverse conditions, such as winter. Laminarin has demonstrated antibacterial properties against a wide range of bacteria in vitro, including pathogenic strains like *E. coli*, *S. Typhimurium*, *L. monocytogenes*, and *St. Aureus* [52,54]. This antibacterial activity extends to purified laminarin extracted from various seaweed species, with a more pronounced effect observed against Gram-negative bacteria [55,56].

When applied in pig nutrition, the inclusion of laminarin-rich extracts in the diet has been associated with reduced populations of *Enterobacteriaceae* and *attaching-effacing Escherichia coli (AEEC)* in the caecum and colon of weaned pigs [57,58]. Moreover, laminarin exhibits prebiotic activity, as demonstrated by an increase in the populations of beneficial *Lactobacillus* species in pig colonic and fecal microbiota following supplementation with both crude and highly purified laminarin-rich extracts [59]. Additionally, investigations in weaned pigs have revealed an increased relative abundance of *Prevotella* spp. following laminarin supplementation, which has been correlated with improved pig performance and maturity [60].

The impact of laminarin supplementation goes beyond microbiota modulation and extends to the production and profiles of short-chain fatty acids (SCFAs) in the gastrointestinal microbiota, particularly affecting butyrate production [35,61]. The SCFAs, including butyrate, are crucial for gut health and have been linked to various physiological benefits. In terms of immunomodulatory activity, dietary supplementation with crude or highly purified laminarin-rich extracts has demonstrated an anti-inflammatory effect on the small intestine and colon of weaned and growing pigs [35]. This anti-inflammatory effect is characterized by the reduced expression of pro-inflammatory cytokine genes, pattern recognition receptors, and the transcription factor NFKB1 [62]. In the colon, laminarin has an immunosuppressive effect, primarily down-regulating genes associated with the Th17 pathway [63]. Furthermore, laminarin-rich extracts have been associated with several performance improvements in weaned pigs, including enhanced final body weight, daily gain, feed intake, and gain-to-feed ratio [63,64]. Additionally, laminarin supplementation has proven effective in reducing diarrhea, particularly in the post-weaning period, as indicated by lower fecal scores in supplemented weaned pigs [57,64]. Importantly, under both hygienic and unsanitary conditions, laminarin-rich extracts have shown promise in reducing the incidence of post-weaning diarrhea and improving daily gains, making them a potential dietary alternative to antibiotic growth promoters and zinc oxide for managing post-weaning diarrhea in pigs [61].

It is essential to acknowledge that the quantitative, structural, and functional variability of laminarin can significantly depend on factors such as extraction methodologies, conditions, and the types of seaweed used [65]. The effectiveness of these β-glucans as bioactive compounds can vary based on parameters such as solubility, molecular weight, and structural characteristics. Although new extraction techniques offer more efficient ways to obtain laminarin from seaweeds, the choice of extraction method and seaweed variety plays a crucial role in determining the quality and properties of the extracted polysaccharides [65]. Understanding these factors is vital for harnessing the full potential of laminarin as a bioactive compound in weaned pig diets.

## 8. The Role of Vitamin D in Nutrition and Immunity

The pivotal role of Vitamin D in swine health is increasingly evident in the context of modern indoor farming practices, which often limit natural sunlight exposure, essential for the endogenous synthesis of this fat-soluble nutrient. Consequently, dietary Vitamin D supplementation becomes crucial to maintaining pig health, supporting a range of physiological processes from bone development to immune function [66].

Although regulatory guidelines, such as those from the European Food Safety Authority [67], stipulate a maximum dietary Vitamin D content of 50 µg/kg/feed, recent research argues that these standards may not suffice in light of intensified agricultural practices [68,69]. Emerging studies propose that enhanced Vitamin D fortification can contribute to improved immune responses, growth rates, and feed conversion efficiency in pigs, challenging the adequacy of current recommendations by authorities like the National Research Council [70]. Furthermore, the interaction of Vitamin D with the gut microbiota—a relationship that is well-established in human research—shows promising implications in swine, where increased levels of 25(OH)D3, particularly in low-calcium and low-phosphorus diets, have been associated with beneficial shifts in fecal microbial compositions [71].

The classical functions of Vitamin D in mineral regulation and bone health are well-known, yet its immunomodulatory capacity is gaining recognition. Vitamin D mediates immune responses through its active form, calcitriol, acting on the Vitamin D receptor (VDR) present in various immune cells, thus influencing both innate and adaptive immunity [72,73]. Notably, Vitamin D is involved in regulating the expression of antimicrobial peptides and the balance between pro-inflammatory and anti-inflammatory cytokine production. Its deficiency is linked to autoimmune diseases in humans, highlighting its systemic relevance [74]. Although more extensively studied in avian species, where Vitamin D is known to exert antioxidative and immune effects [75,76,77,78], the investigation into its effects on pigs suggests similar benefits. For instance, high-dose 25(OH)D3 supplementation has been associated with reduced severity of diarrhea in weaned pigs challenged with pathogens like the porcine epidemic diarrhea virus [79,80]. In light of this evidence, Vitamin D’s function in pig health appears to be multifaceted, offering benefits that extend far beyond its traditional roles. This highlights a clear directive for further research to explore the full potential of Vitamin D in enhancing both the health and performance of the post-weaned pig.

## 9. The Role of Selenium in Nutrition and Immunity

### 9.1. Selenium’s Crucial Role in Pig Health

Selenium is an indispensable trace mineral for swine, playing a crucial role in enhancing immune function, reproductive health, growth, and meat quality. It exercises its biological roles chiefly through selenoproteins, which incorporate the amino acid selenocysteine into their structure at active sites [81]. These selenoproteins, notably glutathione peroxidases (GPXs), thioredoxin reductases (TXNRDs), and iodothyronine deiodinases (DIOs), are essential in modulating the immune system and protecting cells from oxidative harm [82,83]. The dietary form of selenium significantly affects its bioavailability, with organic selenium from sources like enriched yeast showing superior absorption and utilization in the animal’s body compared to inorganic forms such as sodium selenite, which are less bioavailable and can be toxic [84]. Studies have shown that organic selenium more effectively improves selenium status in various tissues and animal products, like colostrum and milk, than inorganic sources [85]. The management of selenium intake is critical, balancing a fine line between deficiency, which can subtly impact growth and reproduction, and toxicity, which may result in severe health repercussions [86].

### 9.2. Selenium’s Impact on Gut Microbiota and Immunity

Emerging research has shed light on the impact of selenium on the gut microbiota. Selenium supplementation is linked to a healthier gut flora composition, increasing beneficial bacteria such as *Lactobacillus* while reducing pathogenic species like *E. coli* [87,88,89,90]. This supplementation is also associated with reduced inflammatory markers and bolstered immune responses, showcasing selenium’s role in modulating inflammation [91,92]. Specifically, in swine, selenium demonstrates the potential to reinforce immunity, particularly under environmental stressors. Providing weaned pigs with selenium-enriched yeast has been shown to enhance growth metrics and immune responses, suggesting a significant role in improving post-weaning resilience [93]. These findings are paralleled in poultry, where selenium has beneficially influenced immune and oxidative stress parameters [94].

At the heart of selenium’s role in gut immunity is its integration into selenoproteins with powerful antioxidant capabilities. Selenoproteins like GPXs and TXNRDs are critical in shielding the gut mucosa from oxidative stress caused by free radicals and reactive oxygen species (ROS) during metabolic activities and immune reactions. By neutralizing these reactive molecules, selenium is vital for the maintenance of gut integrity, therefore averting tissue damage and inflammation that can be induced by oxidative stress [95], a factor crucial for ensuring the health of the gastrointestinal system during the post-weaning period. Selenium is also pivotal in augmenting the gut’s immune response. It is necessary for the proper activation and functioning of immune cells such as T lymphocytes and natural killer (NK) cells, which are key in fighting off pathogens and preserving immune equilibrium in the gut. Selenium has been implicated in enhancing antibody production, vital to the adaptive immune response, thus facilitating the immune system’s capacity to detect and eliminate pathogens, ensuring extensive immune protection in the gastrointestinal tract [96]. Furthermore, selenium’s ability to modulate gut inflammation is paramount. Deficiencies in selenium have been connected to a heightened susceptibility to inflammatory conditions like inflammatory bowel disease (IBD). Selenium exerts anti-inflammatory effects by curtailing the production of pro-inflammatory cytokines such as TNF-α and IL-6, helping to manage inflammation and maintain gut health [97], a factor crucial for improved gut health during the post-weaning period.

The intriguing link between selenium and gut microbiota has been uncovered in recent studies [89,90]. Selenium has been shown to affect the composition and diversity of the gut microbial community, a relationship that has significant implications for gut immunity. The balance and diversity of gut bacteria are instrumental in the immune responses within the gut. Thus, selenium’s influence on the microbiota composition is a critical factor in promoting a balanced and harmonious gut ecosystem, contributing to a robust and responsive gut immune system in the weaned pig.

## 10. Mushrooms as a Source of β-Glucan, Vitamin D and Selenium

Mushrooms, especially the globally consumed *Agaricus bisporus* or white button mushroom, present a unique opportunity in animal nutrition, especially for pigs. The agricultural practice of utilizing mushroom by-products stems from a growing awareness of the sustainability issues related to feed production. These by-products, primarily composed of mushrooms unsuitable for the consumer market, embody a resource for nutrient-rich, bioactive compounds that can significantly contribute to pig nutrition and health [98]. *Agaricus bisporus* is endowed with an array of bioactive compounds that offer considerable health benefits. The presence of β-glucan polysaccharides is well-noted for their role in immune modulation. Moreover, an array of polyphenols, amino acids like ergothioneine, and the presence of chitin, terpenoids, Vitamin D2, and ergosterol broaden the potential health benefits. These compounds are collectively known for their anticancer, antioxidant, antiviral, antimicrobial, antibacterial, antifungal, anti-inflammatory, and immunomodulatory effects [98,99].

Ergosterol plays a particularly interesting role in the nutritional value of mushrooms. When exposed to UV light, ergosterol converts to Vitamin D2, a vital nutrient for various physiological functions, including cell growth, neuromuscular and immune function, and inflammation regulation. This is a crucial feature for indoor-reared pigs that lack natural sunlight exposure, as Vitamin D sourced from mushrooms can help in mitigating deficiency [100,101].

Research has indicated that dietary inclusion of *Agaricus bisporus* can influence the pig gut microbiota favorably and exert an anti-inflammatory effect, although no significant changes were noted in piglet performance [102]. This suggests that the mushrooms may contribute to long-term health benefits that are not immediately apparent in growth metrics [102]. Conversely, Duffy et al. [68] demonstrated that finisher pigs fed with Vitamin D2-enriched mushrooms showed improvements in performance, antioxidant status, and pork color stability, indicating the direct benefits of mushroom-derived Vitamin D2 on pig growth and meat quality.

The biofortification of mushrooms with selenium has become an area of great interest due to selenium’s critical role in pig health. Organic forms of selenium present in mushrooms, such as selenomethionine, have higher bioavailability compared to inorganic selenium sources, such as selenite or selenate, which are commonly used in pig diets. Selenium is a key component of glutathione peroxidase, an enzyme that protects cells from oxidative damage, and it plays a role in thyroid hormone metabolism and immune response [103]. Selenium-enriched mushrooms have been shown to improve pig health by increasing antioxidant capacity, enhancing immune responses, and improving meat quality, which is indicative of selenium’s incorporation into body tissues [104,105]. Furthermore, the supplementation of pig diets with selenium-enriched mushrooms has yielded results such as enhanced gut microbiota, reduced diarrhea scores, and improvements in volatile fatty acids profiles in the caecum [104,105]. These shifts in the gut environment suggest a role for selenium-enriched mushrooms in promoting a gut microbiota that is favorable for pig health, potentially reducing the need for medical interventions and contributing to the resilience of the animals [104,105] Given these multi-dimensional benefits, the integration of mushroom by-products into pig diets extends beyond mere nutrient supplementation.

## 11. Synergistic Effects of β-Glucan with Casein Hydrolysates

Casein hydrolysates, which are derived from milk protein casein, are gaining attention as potential alternatives to antibiotic growth promoters in pig nutrition [106]. They are valued for their high nutritive content and the presence of bioactive peptides [107,108]. These bioactive peptides are naturally embedded within the structure of casein and can be released through enzymatic hydrolysis during digestion or food processing [107,108]. Extensive research has demonstrated the significant bioactivity of casein hydrolysates in various experimental settings, including in vitro, ex vivo, and in vivo studies [109,110]. However, a common challenge with bioactive compounds is their often-limited bioavailability in vivo, which can restrict their effectiveness as health-promoting agents [111].

One potential issue is that the bioactivity of casein hydrolysates may be compromised in the stomach during digestion. To address this concern, microencapsulation techniques can be employed to protect these bioactive compounds and enhance their bioavailability [106,112]. For example, studies have shown that microencapsulation using substances like yeast β-glucan can preserve the bioactivity of casein hydrolysates in vivo [106,112]. It is worth noting that β-glucans themselves have been extensively studied for their antioxidant, immunological, and anti-inflammatory effects [113,114,115], but research on the use of casein hydrolysates in pig diets is relatively limited. Some studies have explored their potential benefits. For instance, piglets supplemented with yeast β-glucan and casein hydrolysate exhibited reduced inflammation, characterized by the upregulation of tight junction protein CLDN3 and the downregulation of pro-inflammatory cytokine genes [116], factors crucial for improved gut health during the post-weaning period. Furthermore, in post-weaning pigs, the combination of casein hydrolysate and yeast β-glucan was found to improve gastrointestinal function and health [106]. These findings suggest that the use of casein hydrolysates in combination with microencapsulation techniques, such as those involving β-glucans, holds promise for enhancing the bioavailability and effectiveness of these bioactive compounds in improving pig health and performance.

## 12. The Benefits of Incorporating β-Glucans into Sow Diets

The development of the GIT and immune system in piglets is profoundly influenced by maternal factors during gestation and lactation. Supplementing the diets of gestating and lactating sows has the potential to positively impact the health of piglets, particularly during the pre-weaning phase, which may lead to improved post-weaning performance [24]. The GIT colonization in piglets starts immediately post-birth, with bacteria from the sow and the environment crucial in this process [117]. The microbiota composition of piglets can be influenced by altering the sow’s diet to encourage beneficial bacteria and decrease pathogenic species, potentially reducing piglet susceptibility to PWD [118,119]. The sow’s vaginal and fecal microbiota are significant contributors to the piglet’s intestinal microbiota. Crespo-Piazuelo et al. [120] showed that sows supplemented with a probiotic containing *Bacillus altitudinis* resulted in piglets displaying fecal shedding of the probiotic strain, suggesting a vertical transmission of beneficial bacteria. Additionally, laminarin and fucoidan from seaweed extracts given to gestating sows reduced Enterobacteriaceae levels in sow feces and decreased colonic Escherichia coli in piglets at weaning [119,121]. Furthermore, yeast β-glucan combined with casein hydrolysate in sow diets during late pregnancy has been linked to a more favorable fecal microbiota composition, with increases in beneficial bacteria like Lactobacillus [116]. Such piglets weaned from these supplemented sows had increased villus height in the duodenum and increased villus height to crypt depth ratio in the jejunum, as well as a decreased expression of the pro-inflammatory cytokine genes, the tight junction gene *CLDN3* and the mucin gene *MUC2* in the duodenum and jejunum.

The quality of colostrum and milk is vital for delivering antimicrobial and immune-enhancing properties to piglets [118]. Colostrum intake is essential for stimulating intestinal growth and function, facilitating the absorption of immunoglobulin G (IgG) for systemic immunity, and providing energy for thermoregulation [122,123,124]. Colostrum and milk contain immunoglobulins and other antimicrobial compounds that support the establishment of a beneficial commensal microbiota [118]. The IgG, abundant in colostrum, decreases after birth, but its early presence is critical for protection against infections during weaning [125,126]. Similarly, IgA provides a crucial defense against GIT pathogens and plays a role in preventing PWD [127]. Dietary supplementation of gestating and lactating sows with β-glucans and other bioactive compounds can enhance immunoglobulin concentrations in colostrum and milk, potentially improving piglet health outcomes [128,129,130]. Piglets from sows fed with laminarin and fucoidan-rich diets showed improved immune function, such as enhanced leukocyte phagocytosis capacity (Leonard et al., 2010) and increased resistance to ETEC infections [119].

In summary, the strategic dietary supplementation of sows with β-glucans and other bioactive compounds can significantly influence piglet GIT colonization, immunity, and resilience against post-weaning gastrointestinal challenges. Through these nutritional interventions, it is possible to reduce the presence of pathogenic bacteria, enhance immune function, and improve piglet growth and health outcomes.

## 13. Conclusions

The multifaceted roles of β-glucans, derived from diverse sources such as yeast, mushrooms, cereals, and seaweeds, are becoming increasingly recognized for their potential in swine nutrition. These polysaccharides, through their immunomodulatory, antimicrobial, and gut health-promoting activities, offer a natural alternative to traditional feed additives. Yeast-derived β-glucans, with their robust impact on immune cell activation, have shown promising effects on the growth performance and gut microbiota composition of weaned pigs, enhancing the presence of beneficial bacteria while suppressing pathogenic strains. Similarly, mushroom-derived β-glucans, along with an array of other bioactive compounds, contribute to antioxidative capacity and overall animal well-being, with added benefits from Vitamin D and selenium fortification. Laminarin from seaweed adds to the complexity of β-glucans in swine diets by providing distinctive antibacterial and prebiotic effects that could support intestinal health and improve post-weaning growth metrics. Moreover, the incorporation of β-glucans into sow diets may impart long-term health benefits to piglets, emphasizing the importance of maternal nutrition on offspring development.

Although the benefits of β-glucans are evident, the picture is nuanced. It is essential to select appropriate sources and doses of β-glucans and to consider synergy with other compounds, such as casein hydrolysates, for maximum efficacy. Furthermore, the influence of Vitamin D and selenium, particularly from mushroom sources, extends the potential health benefits for post-weaned pigs, reinforcing the need for a strategic, well-balanced approach to dietary supplementation. By enhancing immunity, promoting healthy gut microbiota, and improving growth and resilience, β-glucans stand as a significant contributor to the advancement of sustainable and productive pig nutrition practices post-weaning.

## Data Availability

Not applicable.
